# Comparative profiling of microRNAs in the winged and wingless English grain aphid, *Sitobion avenae* (F.) (Homoptera: Aphididae)

**DOI:** 10.1038/srep35668

**Published:** 2016-10-20

**Authors:** Xiangrui Li, Fangmei Zhang, Brad Coates, Yunhui Zhang, Xuguo Zhou, Dengfa Cheng

**Affiliations:** 1 State Key Laboratory for Biology of Plant Diseases and Insect Pests, Institute of Plant Protection, Chinese Academy of Agricultural Sciences, Haidian District, Beijing 100193, China; 2 Xinyang Agriculture and Forestry University, Xinyang, Henan 464000, China; 3 United States Department of Agriculture, Corn Insects & Crop Genetics Research Unit, USDA-ARS, Ames, IA 50011, USA; 4 Department of Entomology, University of Kentucky, Lexington, KY 40546, USA

## Abstract

MicroRNAs (miRNAs) are short single-stranded non-coding RNAs that regulate gene expression, particularly during development. In this study, 345 miRNAs were identified from the English green aphid, *Sitobion avenae* (F.), of which 168 were conserved and 177 were *S. avenae*-specific. Quantitative comparison of miRNA expression levels indicated that 16 and 12 miRNAs were significantly up-regulated in winged and wingless *S. avenae* small RNA libraries, respectively. Differential expression of these miRNAs was confirmed by real-time quantitative RT-PCR validation. The putative transcript targets for these candidate miRNAs were predicted based on sequences from a model species *Drosophila melanogaster* and four aphid species *Acyrthosiphon pisum, Myzus persicae, Toxoptera citricida,* and *Aphis gosspii*. Gene Ontology and KEGG pathway analyses shed light on the potential functions of these miRNAs in the regulation of genes involved in the metabolism, development and wing polyphenism of *S. avenae*.

The transcription of tRNAs, rRNAs and mRNAs have long been known to function in the central dogma of synthesizing functional proteins from nucleic acid information, but a large class of initially characterized non-coding RNAs (ncRNAs) may play important roles in regulating the expression of protein coding genes. Nucleic acid-protein complexes perform an array of crucial cellular functions. For example, the U1 small nuclear RNA (snRNA) functions to regulate transcription initiation^1^, the spliceosome guides intron splicing activities^2^, and the ribosome facilitates protein synthesis from mRNA templates^3^. This known cellular repertoire of functional RNAs has expanded. Specifically, RNA interference (RNAi) pathway involves the use of short anti-sense guide RNAs bound to the protein Argonaut within the RNA Inducing Silencing Complex (RISC) which binds the sense strand of cellular RNA thereby targeting them for degradation by the endonuclease Dicer. These short guide RNAs, microRNAs (miRNAs) and small interfering RNAs (siRNAs), are derived from the degradation of dsRNAs and short hairpin RNAs by Dicer. Although entering a unified pathway, miRNAs and siRNAs carry out different functions by respectively regulating endogenous gene expression and degrading invading foreign RNAs^4^. The biogenesis of miRNAs occur from secondary structure elements formed by transcribed ncRNAs as well as stem-loops formed by spliced intron and gene encoding transcripts themselves, and are collectively termed primary RNAs (pri-RNAs). These pri-RNAs are cleaved into shorter 70 base hairpin precursor RNAs (pre-RNAs) within the nucleus by the ribonuclease III, Drosha, in complex with the RNA binding protein, Pasha^5^. Pre-RNAs are also known to be generated directly from spliced intronic RNAs called mirtrons^6^. Following transport into the cytoplasm, pre-RNA terminal loops are cleaved by the ribonuclease, Dicer, to form a short 21 to 22 bp miRNA product with a 2 nucleotide overhang. The degradation of the passenger strand generates a functional ssRNA guide within the RISC complex^7^.

The miRNA guide stand directs the RISC complex by imperfect complementary base pairing mainly to 3′ untranslated regions (UTRs) of target mRNAs, as well as to 5′ UTRs and exons^8^. Despite the relaxed complementarity allowed along the 21 to 22 bp of the guide RNA/target RNA duplex, a near perfect match is required in the “seed” region located from positions 2 to 7 of the guide miRNA is believed to be involved in target specificity^9^. This miRNA-mediated binding of RISC is speculated to result in post-transcriptional repression by mRNA degradation, translational repression by blocking initiation factor binding, inhibition of elongation factor progression, or causing premature termination^10^. In contrast, miRNAs can upregulate gene transcription by potentially reversing the effects of repressive miRNAs^11^. Studies have demonstrated that miRNAs regulate a large fraction of mammalian protein-coding gene^12^, such that miRNAs may be key mediators in a range of developmental and physiological pathways including embryonic development^13^, tissue differentiation and apoptosis^14^, cell proliferation^15^, and morphogenesis^16^.

Studies have also shown that miRNAs are regulators of pattern formation necessary for insect wing development. Specifically, the Bantam miRNA guide directs RISC for targeted degradation of mRNAs for the gene *enabled*, the latter of which is required for pattern formation during *Drosophila melanogaster* development. *Bantam* miRNA levels are in turn repressed in wing imaginal discs by the expression of *Notch*, which subsequently alleviates repression of *Enabled* and leads to the formation of doral-ventral polarity in developing embryos^17^. Within wing imaginal discs of these developing *D. melanogaster* embryos, the *Let-7* miRNA directs the specific timing of cellular division^18^. Indeed, the depletion of *Let-7* and *miR-100* reduced wing size and generated malformed vein patterning in the German cockroach, *Blattella germanica*^19^. Analogously, the loss-of -function *miR-9a* mutant leads to wing malformations due to dysregulation of the number of neuronal precursor cells during *Drosophila* development^20^. Surridge and colleagues^21^ also described the specific expression of *miR-193* and *miR-2788* between 24 and 72 hours post-pupation in the postman butterfly, *Heliconius melpomene*, and suggested their potential role in wing development. The two insulin receptors, *InR1* and *InR2*, were suggested to regulate the development of alternative wing morphs in the migratory brown planthopper *Nilaparvata lugens*^22^. In the pea aphid, *Acyrthosiphon pisum*, comparisons of miRNA abundance among parthenogenetic females producing parthenogenetic females (virginoparae), parthenogenetic females producing sexual individuals (sexuparae), and sexual females only (oviparae), showed a phase-specific expression of miRNAs^16^.

The grain aphid, *Sitobion avenae* (F.), is a destructive pest of wheat crops that is distributed worldwide. Adults show two different morphological variants, winged and wingless that exists among clonal genetically-identical individuals produced from parthenogenic females. This polyphenism is observed in response to the environment, and results in isogenic offspring in the subsequent generation showing difference in wing development^23^. In this study, high-throughput sequencing was performed on short RNA libraries constructed from winged and wingless adults, from which we aim to detect and quantify the levels of these putative miRNAs in the *S. avenae*, as well as identify potential regulatory effect by target transcripts involved in wing development in *S. avenae*. These findings suggest that a specific set of miRNAs are potentially involved in regulating genes that direct wing development and provide resources for future hypothesis-driven research.

## Results

### RNA isolation, small RNA library construction and Illumina sequencing

**Figure 1:**
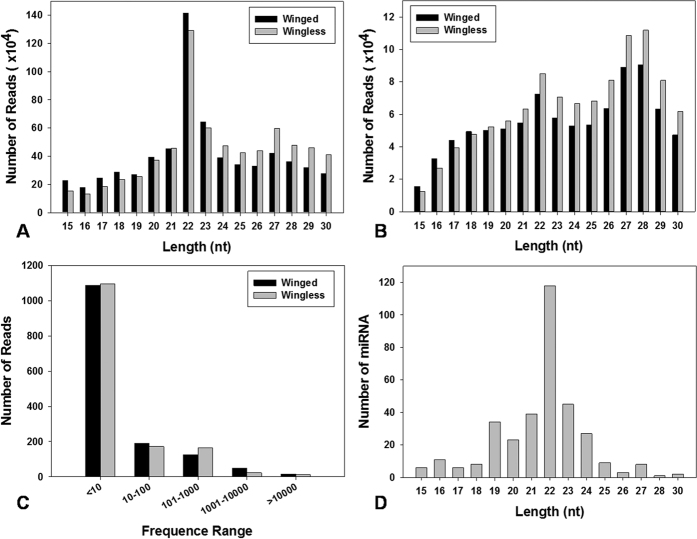
Characterization of small RNA sequences from *S. avenae* deep sequencing. Length distribution of raw reads (**A**) and mappable reads (**B**), distribution of frequencies on read counts (**C**) and number distribution of small miRNAs (**D**).

**Figure 2:**
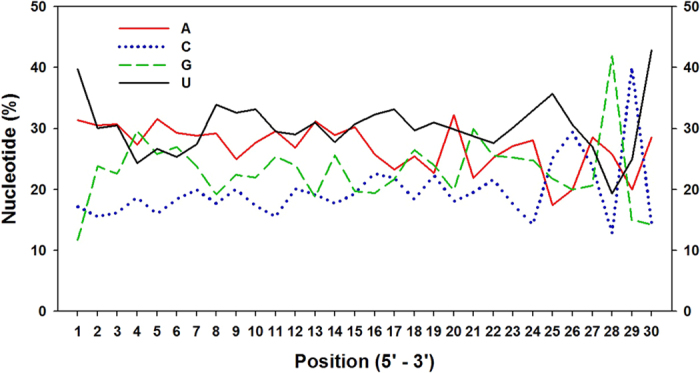
Nucleotide bias of predicated *S. avenae* miRNAs.

A total of 13,760,466 and 15,594,991 raw reads were obtained from winged and wingless sRNA libraries, respectively. The lengths of *S. avenae* sRNAs ranged from 10 nt to 30 nt, with 22 nt size comprising 21.63% and 18.58% of the total reads from winged and wingless libraries, respectively (Fig. 1A). After filtering out adapter and low quality sequences, and removal of rRNAs, tRNAs, snRNAs and snoRNAs, and repeat regions, 887,980 (49.95% of total) and 1,033,351 (50.02%) unique reads, respectively, remained from winged and wingless libraries (Table 1). In both libraries, the length distribution of unique filtered reads showed a bimodal distribution (Fig. 1B), wherein the peak at 22 nt constituted 8% of the total reads. A second size class was observed at the 27–28 nt size range (20%) which likely might represent *S. avenae* piRNA-like sRNAs. Furthermore, a small number of sRNAs had more than a thousand reads, whereas the majority had fewer than ten copies in the library (Fig. 1C). *Sitobion avenae* sRNAs exhibited a strong bias for the nucleotide U at 5′ (39.73%) and 3′ ends (42.86%), and a paucity of G at 5′ end (11.7%; Fig. 2).

### Sequence analysis and miRNA predication

**Figure 3:**
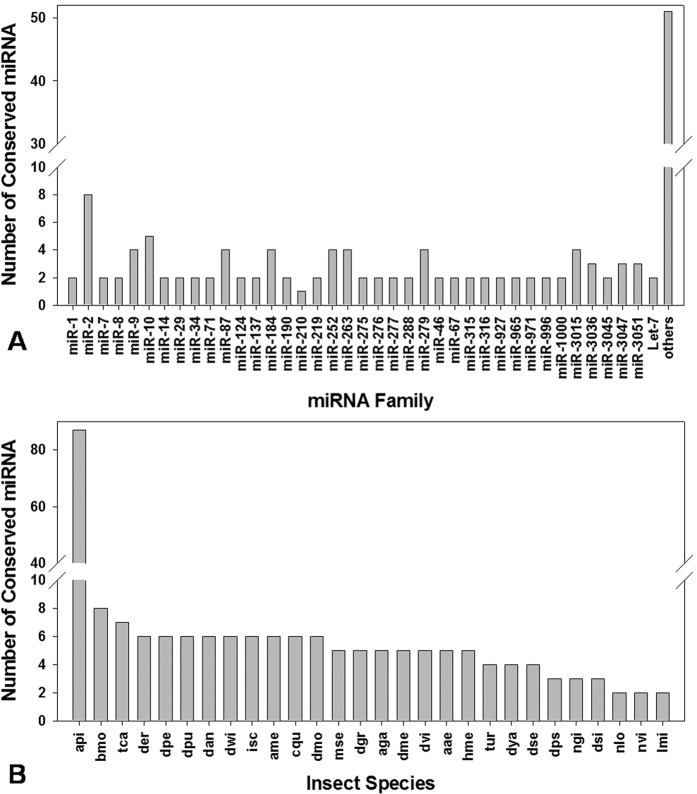
Characterization of conserved *S. avenae* miRNAs. (**A**) Number of identified conserved miRNAs in each miRNA family; (**B**) conservation profile of identified miRNAs in insect species. api: *Acyrthosiphon pisu*m; bmo: *Bombyx mori*; tca: *Tribolium castaneum*; der: *Drosophila erecta*; dpe: *Drosophila persimilis*; dan: *Drosophila ananassae*; dwi: *Drosophila will*; isc: *Ixodes scapularis*; ame: *Apis mellifera*; cqu: *Culex quinquefasciatus*; dmo: *Drosophila mojavensis*; mse: *Manduca sexta*; dgr: *Drosophila grimshawi*; aga: *Anopheles gambiae*; dme: *Drosophila melanogaster*; dvi: *Drosophila virilis*; aae: *Aedes aegypti*; hme: *Heliconius Melpomene*; tur: *Tetranychus urticae*; dya: *Drosophila yakuba*; dse: *Drosophila sechellia*; ngi: *Nasonia giraulti*; dsi: *Drosophila simulans*; nlo: *Nasonia longicornis*; nvi: *Nasonia vitripennis*; lmi: *Locusta migratoria.*

Based on the read length distribution, 22 nt-long miRNAs represented ~34% of the total miRNA species within sRNA libraries (Fig. 1D). Queries of these putative *S. avenae* miRNA reads against the *A. pisum* genomes and all insect miRNAs in miRBase resulted in the identification of 168 conserved sequences (Table S1). Additionally, the query of filtered *S. avenae* reads to the collection of mature miRNAs in the miRBase database identified 39 conserved miRNA sequence families (Fig. 3A). The *miR-2* family was predicted to contain the most family members (*n* = 8), followed by *miR-10* (*n* = 5), and *miR-87*, *-184*, *-252*, *-263*, *-279*, *-9*, *-3015* (*n* = 4). These conserved miRNAs were shared across insects, with the majority being shared with known miRNAs from *A. pisum*, and a lesser extent towards putative orthologs from other insects (Fig. 3B). These results suggest that the seed region of mature miRNA sequences (base positions 2 to 8 from the 5′ end) are highly conserved across insects, whereas the 3′ tail and central nucleotide positions tend to be more divergent. For example, *Let-7* and *miR-7,* comparatively, shares identical seed sequences across all insects (Fig. S1; Fig. S2).

A total of 177 potentially novel miRNAs were also identified and given the prefix ‘PC’ (predicted candidate) in the nomenclature we adopted (Table S2). These putatively *S. avenae* specific miRNAs were mapped to the *A. pisum* genome sequences in spite of having no detectable homology to any known insect pre-miRNAs in miRBase. Results from Mfold predicted that all *S. avenae* miRNAs precursor sequences form stem-loop hairpin secondary structures (≤18 kcal/mole^24^), and a subset of these predicted structures are shown (Fig. S3).

### Differential miRNA expression between winged and wingless morphs

**Figure 4:**
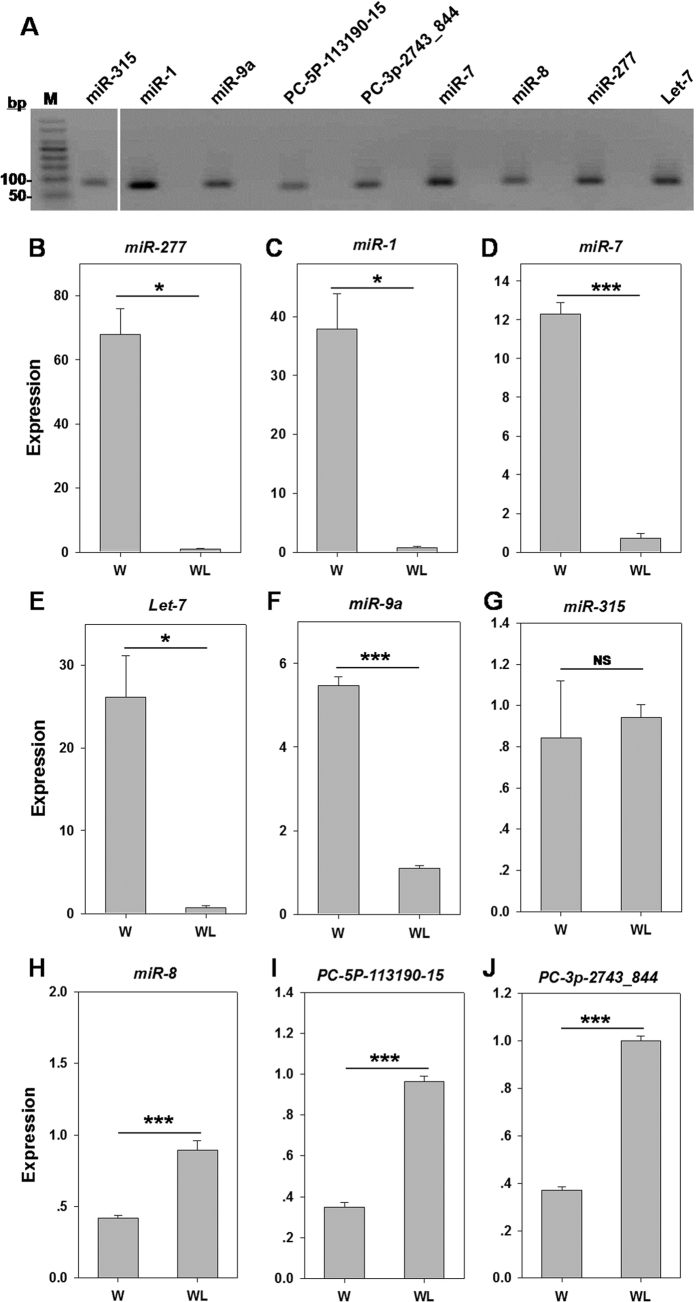
RT-qPCR validation of miRNAs potentially involved in *S. avenae* wing development. (**A**) cDNAs from the winged *S. avenae* were used for template. Lane 1: 100 bp ladder marker; Lane 2: *miR-315*; Lane 4: *miR-1*; Lane 6: *miR-9a*; Lane 8: *PC-5p-113190_15*; Lane 10: *PC-3p-2743_844*; Lane 12: *miR-7*; Lane 14: *miR-8*; Lane 16: *miR-277*; Lane 18: *Let-7*. The other uneven lanes were negative controls for each target miRNA. The relative miRNA expression, including *miR-277* (**B**), *miR-1* (**C**), *miR-7* (**D**), *Let-7* (**E**), *miR-9a* (**F**), *miR-315* (**G**), *miR-8* (**H**), *PC-5p-113190_15* (**I**), and *PC-3p-2743_844* (**J**) at two wing morph was normalized to the wingless adult. W: winged adult; WL: wingless adult. *P < 0.05; **P < 0.01; ***P < 0.001. Full-length gel is presented in Supplementary Figure S4.

Based on normalized differences in Illumina read counts, 28 out of 345 *S. avenae* miRNAs (8.5%) showed significant differences in their expression level between winged and wingless morphs, including 12 up-regulated and 16 down-regulated miRNAs (Table 2). The RT-PCR amplified products for seven conserved miRNAs (*Let-7*, *miR-1*, *miR-7*, *miR-277*, *miR-8*, *miR-9a* and *miR-315*) and two novel miRNAs (*PC-5p-113190_15* and *PC-3p-2743_844*) showed a single band in the expected size (60–100 bp) (Fig. 4A and S4). Subsequent analysis of real-time RT-qPCR results confirmed that the expression of five miRNAs were significantly lower in wingless compared to winged *S. avenae*. The highest RT-qPCR Log_2_ fold-changes were observed in *miR-277* and *miR-1* that respectively showed reductions amongst wingless adults of 73.2- (Fig. 4B) and 47.5-fold (Fig. 4C). Similarly, the expression of *miR-7*, *Let-7,* and *miR-9a* were 17.1-, 36.5-, 4.9- fold lower in wingless adults, respectively (Fig. 4D–F). In contrast, *miR-8*, *PC-5p-113190_15*, and *PC-3p-2743_844* were up-regulated in wingless adults (Fig. 4H–J). All the above comparisons were significant (*P* ≤0.036), only *miR-315* showed no significant difference between the two morphs (t = −0.60, *P* = 0.58) (Fig. 4G). Overall, RT-qPCR results were consistent with RNA-seq analyses, except *miR-9a*. Although the fold changes of their expression level were different, the trend was, for the most miRNAs, the same.

### MicroRNA target prediction

**Figure 5:**
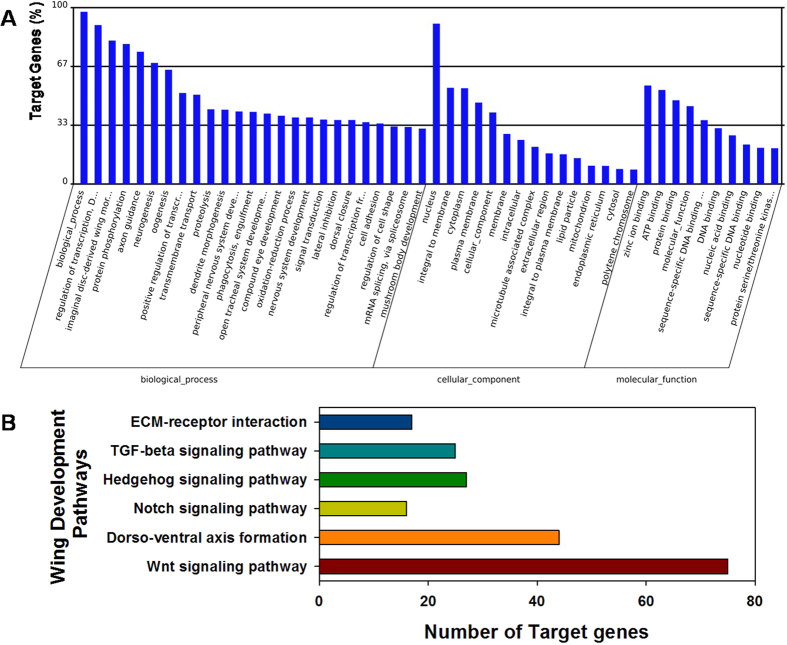
Histogram presentation of GO annoation and KEGG pathway for identified miRNAs in *S. avenae*. (**A**) Gene Ontology classification based on level two. The results are summarized in three main categories: biological process, cellular component and molecular function. The y-axis indicates the number of genes and its proportion in a category; (**B**) number of target genes joining wing development by KEGG analysis.

Using the 28 differentially expressed *S. avenae* miRNA sequences, Target Scan predicted 17,253 putative targets for these miRNAs within the UTRs of transcripts from the model insect *D. melanogaster* and aphid species *A. pisum, M. persicae*, *T. citricida* and *A. gosspii* (Table S3). This was performed due to the lack of transcript and genome sequence data for *S. avenae*. Functional annotation of these putative target genes by GO enrichment predicted potential involvement in various biological processes, cellular components and molecular functions, including development processes (Table S4). A total of 50 GO terms were identified for predicted miRNA target genes based on GO level 2 (Fig. 5A), including genes involved in wing development processes (e.g. GO: 0035311: wing cell fate specification with 2 targets; GO: 0007472: wing disc morphogenesis with 18 targets; GO: 0007476: imaginal disc-derived wing morphogenesis with 334 target genes).

KEGG enrichment analysis predicted that genes within 124 metabolic pathways may be affected by miRNA targeting (regulation). These pathways include genes in *Wnt* (*n* = 75), *Notch* (*n* = 16), *Hedgehog* (*n* = 27), and *TGF*-beta signaling pathways (*n* = 25), and gene products involved in extra cellular matrix (*ECM*)-receptor interaction (*n* = 17) and dorso-ventral axis formation (*n* = 44; Fig. 5B). This transcript target prediction to orthologs from related insects indicated that *S. avenae* miRNAs which show significant differential expression between winged and wingless morphs may bind and facilitate the post-transcriptional regulation of various biological pathways, including wing development (Table S5).

## Discussion

Ever since their initial characterization, research into the cellular roles of miRNAs has led to their description as crucial factors in the regulation of transcript abundance and therefore gene expression. MiRNAs are described among a diverse set of organisms including vertebrates, plants, arthropods and viruses (ftp://mirbase.org/pub/mirbase/CURRENT). Moreover, processes in insect development and metamorphosis are regulated by miRNAs through either degradation of target mRNA or inhibition of translation^14^,^19^,^20^,^25^. For example, the product of *Decapentaplegic* (*Dpp*) is excreted from *D. melanogaster* embryonic cells and forms a gradient that is required for proper pattern formation during development, and furthermore regulates the expression of other pattern formation genes^26^. Cell differentiation of the wing is directed by signals from receptors for both *Dpp* and epidermal growth factor (*EGF*), which in turn requires a gradient of *vein* (*vn*) and *wingless* (*wg*) gene products that respectively are directed by *EGF* and *Wnt* ligands^27^. The expression of these diffusible ligands, as well transcription factors that coordinate gene expression are crucial for the direction of wing formation in *D. melanogaster*^28^. Analogously, miRNAs have also been implicated in modulating gene expression during wing development^29^, and includes the roles of *miR-7*^30^, *miR-iab-4*^31^, and *miR-2a*^32^ (Table 3).

In the current study, we obtained 12.0 million reads from sRNA libraries wherein the length distribution of mature miRNAs in two *S. avenae* morphs showed a bimodal distribution of unique reads, 22 nt and 27–28 nt. The 22nt reads were shown to represent miRNAs as was found previously^33^, and is consistent with the size common for miRNAs resulting from Dicer digestion as well as analogous for the length distribution observed in *D. melanogaster*^34^, *A. pisum*^16^and *Spodoptera litura*^35^. The shorter sequences may be endo-siRNAs, and the longer may be piRNAs which interact with PIWI proteins and repress the expression of selfish genetic elements such as transposons^36^. These results show that miRNA genes have length diversity, which depends strongly on the asymmetric structural motifs present in precursor hairpins^37^.

The miRNA sequence composition at 5′ ends showed a strong preference for U against G at the first position^33^. Analogously U was the most and C the least frequently observed among miRNA sequences from *S. avenae*, at both 5′ and 3′ end (accounts for 39.73% and 42.86%, respectively; Fig. 2), which is similarly observed among other organisms^16^,^35^. In our analysis, we also predicted that A and U are the most common at positions 2, 3, and 5–8, the latter which corresponds to the “seed sequences” that are known to play a critical role in mature miRNAs targeting of mRNAs for translational inhibition or mRNA cleavage^38^. This base composition may likely play a role in the binding properties of miRNAs to their target mRNAs^33^.

In total, we obtained 168 conserved and 177 *S. avenae*-specific miRNAs *in silico* using *A. pisum* genome as a reference for comparison. The frequency of short nucleotide reads generally are highly representative of relative abundance and were used to estimate the expression level of miRNAs^39^,^40^. Highly expressed miRNAs would be likely to have a large number of sequenced reads. Some of the miRNAs identified in this study had more than thousand reads, while we found that frequencies of many miRNAs were extremely low in our library (<10 reads), which was consistent with previous conclusions suggesting that these miRNAs might be express at low levels in the specific cell types or in limited physiological processes^41^. Our data shows that *MiR-276* was the most highly expressed miRNA in both morphs, wherein it represented 473,103 reads in the winged adults and 369,649 reads in the wingless adults. To date, *miR-276* has been identified in over 34 organisms and may have a critical role in development among various organisms, but the function of this miRNA in insects remains unknown^40^. The 42 out of 168 conserved miRNAs were highly abundant (>1000 reads), while the number of reads for 2 of the 177 novel miRNAs were more rare (<1000 reads). Differences in miRNA abundance may be related to the different roles that miRNAs may play in insect development, as was suggested in *A. pisum* where increased expression of a miRNA was proposed to indicate a possible role in the temporal or spatial repression of specific target mRNAs^16^. The conserved *S. avenae* miRNAs were placed into 39 miRNA families, with each miRNA family potentially having different regulatory functions^37^, or influencing the expression of genes involved in different morphs^40^. Different members in a given miRNA family varied on the estimated number of reads, suggesting that individual miRNA family members could regulate target genes coordinately and shared common functional relationships^42^. A total of 12 up-regulated and 16 down regulated miRNAs were predicted by the IDEG6 program, indicating that these miRNAs may be involved in diverse functions in the two wing morphs.

Mature insect miRNA sequences are highly conserved among different species. Most of the homologous miRNAs share the same “seed region”, the 5′ region which is important for mRNA target recognition in almost all the insects^38^. For example, *miR-7* and *let-7* were high conserved at the 5′ end of the mature sequence in comparison to other insects. Notably, the variation was found at the tail or the middle position, especially for last three nucleotides, which was in line with Wheeler’s observations^43^.

Deep sequencing, in conjunction with RT-qPCR validation, provides an effective means for the identification of miRNAs, as demonstrated in *B. mori*^33^, *Locuta migratoria*^40^, and *A. pisum*^16^, as well as in the current study. Specifically, Legeai *et al*.^16^ reported that 17 miRNAs from *A. pisum* shows significant differences in their steady-state levels between two morphs, in which *Let-7* and *miR-100* with similar expression patterns were up-regulated and *miR-2a* was down-regulated between oviparae and the two other parthenogenetic morphs (virginoparae and sexuparae). This study analogously shows that *Let-7* and *miR-100* are significantly down-regulated among wingless *S. avenae* (Table 2, Fig. 4E), suggesting that *Let-7* and *miR-100* may be import in the differentiation of aphid morphotypes. Other miRNAs have been shown to affect lifespan by post-transcriptional silencing of mRNAs, where loss of *Let-7* in mutant *Drosophila* have a reduced lifespan and show degrees of neurodegeneration^44^. Additionally, expression of *miR-277* shortens *Drosophila* lifespan and is synthetically lethal with reduced insulin signaling^45^. This study shows that *miR-277* and *Let-7* are significantly down-regulated among wingless *S. avenae* (Table 2, Fig. 4B,E), which may be related to the generally observed increased lifespan of winged compared to wingless *S. avenae*. This increased lifespan in aphids could potentially be related to time requires involved in long distance migrations. Interestingly, *miR*-9a was also highly expressed in winged *S. avenae* which has been reported to control the generation of sensory organs in *Drosophila* adult wing imaginal discs^46^, but any potential effect any differences in sensory organ development or function within winged aphids remains unknown. Orthologs of the differentially regulated *S*. *avenae miR-1* and *miR-315* were previously associated with flight behaviors. Specifically, *miR-1* is a muscle-specific miRNA that in conjunction with *miR-315* is differentially regulated in gregarious compared to solitary locusts, and although their functions remain uncertain, Wei *et al*.^40^ hypothesized these miRNAs may affect thorax muscle and the wing functions.

Many studies analyze the function of miRNA during insect wing development^19^,^20^,^21^, wherein *miR-315* is known to be a potent activator of Wingless signaling in *Drosophila*^47^. Evidence indicate that the loss of *miR-7* function results in a reduction of wing size and produces smaller wing cells compared to wild types^30^, and decreases in *miR-277-3p* levels were associated with the reduced size of mutant imaginal discs^48^. Moreover, *miR-8* influences cell survival and epithelial organization in *Drosophila* wings^49^ and the *miR-9a* prevents apoptosis during *Drosophila* wing development. Orthologs of *D. melanogaster* genes involved in wing development are annotated in *A. pisum* genome^50^, of which some are differentially regulated between winged and wingless green peach aphids, *M. persicae*^51^.

Based on GO enrichment and KEGG analyses, the putative orthologous targets of differentially expressed *S. avenae* miRNAs may be involved in wing cell fate (2 gene targets), wing disc morphogenesis (18 targets), imaginal disc-derived wing morphogenesis (334 targets), and several additional pathways (Wnt signaling pathway, dorso-ventral axis formation, Notch signaling pathway, Hedgehog signaling pathway, TGF-beta signaling pathway and ECM-receptor interaction; Fig. 5). Since pathway analysis suggests that putative transcript targets of 28 differentially expressed miRNAs may be associated with wing development, it is enticing to hypothesis that these miRNAs could potentially participate in *S. avenae* wing development. Generating genomic tools, such as development of transcriptome or full genome resources, are required for future investigations of *S. avenae* miRNA roles in key developmental, cellular or behavioral processes.

Regardless, the predicted differences in the abundance of 28 miRNAs between *S.avenae* winged and wingless morphs could potentially be involved in the observed wing polyphenism. Caution should be taken with regards to the interpretation of these results from cross-species analysis since we were likely limited to the discovery of targets for conserved miRNAs. Granted, many miRNAs are highly conserved across evolutionary boundaries^52^, suggesting that the mRNA targets of these highly conserved miRNAs may also be retained across species. In contrast, many taxon-specific miRNAs exist^53^, including 177 *S. avenae* specific miRNAs, suggesting that the likelihood of accurate prediction of putative targets for such miRNAs may be diminished as evolutionary distance with model species increase. Undoubtedly, additional future research is required to identify the targets of *S. avenae* miRNA and unravel their effects on morphotype development. Insulin/insulin-like growth factors, part of an evolutionarily conserved signaling pathway that modulates wing dimorphism in *N. lugens*^22^, might be one of these targets. These efforts may also reveal new insight into the interactions between different aphid miRNAs and duplicates of the RISC components *dicer* and *argonaute* found amongst the Aphidae, including *S. avenae*^54^, where these paralogs of the RISC complex are differentially regulated between wing morphs.

## Materials and Methods

### Aphid colony maintenance

*Sitobion avenae* adults were collected from wheat fields in Langfang, Hebei Province, China (39°30′42′′N, 16°36′7′′E) in 2012, and maintained on 15 cm wheat seedlings at 20 °C, 60% RH, and L:D 16:8 h photoperiod in an RXZ-380B environmental cabinet (Nb-Jn Instrument Factory, Ningbo, China). A separate isogenic aphid colony was generated from a single wingless adult female, and the resulting clonal full-sibling *S. avenae* adults were separated into winged and wingless groups. Individuals were dissected under a stereomicroscope (Nikon SMZ1500, Japan) to remove abdomens to avoid pseudo embryos, and the remaining tissues were immediately transferred into 1.5 mL microcentrifuge tubes floating on liquid nitrogen. Pooled samples of winged and wingless *S. avenae* were stored at −80 °C.

### RNA isolation, small RNA library construction and Illumina sequencing

Total RNAs were isolated from pooled winged and wingless *S. avenae* samples using Trizol reagent (Ambion, USA) according to manufacturer’s instructions. The resulting total RNA quantity was estimated on a NanoDrop spectrophotometer (ND-2000, USA), and by densitometry following denaturing agarose gel analysis. RNA integrity was further assessed using the Bioanalyzer 2100 (Agilent, CA, USA). Total RNA of each sample was size-fractionated on 15% TBE polyacrylaminde gel. Small RNA (sRNA) populations of 15–50 nt were extracted, purified, and ligated to 3′ chimeric oligonucleotide adapters (5′-TGG AAT TCT CGG GTG CCA AGG -3′) and 5′ (5′-GTT CAG AGT TCT ACA GTC CGA CGA TC -3′) using T4 RNA ligase (Illumina, USA). Ligation reaction products were used as template for synthesize of single-stranded cDNA with SuperScript II Reverse Transcriptase (Illumina, USA), and subsequently PCR amplified using Illumina’s primer set for 15 cycles. Amplified cDNA products were gel purified and sequenced on an Illumina HiSeq 2500 (Illumina, San Diego, CA, USA) at LC Sciences (Houston, TX, USA) using the LC Bio service (Hangzhou, China).

### Bioinformatics analysis and miRNA prediction

Proprietary software, ACGT101-miR, was used to analyze Illumina HiSeq 2500 sequencing data generated from *S. avenae* winged and wingless small RNA libraries at LC Science (Houston, TX, USA). In brief, raw reads were filtered to remove the adaptor sequences and contaminated reads (including trim 3′ adapter), and then for reads <15 nt and low-quality reads with Phred quality score (Illumina 1.8+) <20. The remaining high quality reads were initially mapped to RepBase (http://www.girinst.org/repbase/) to identify putative transposon-derived miRNAs, then mapped to the ncRNA database (including rRNAs, tRNAs, snRNAs, and snoRNAs) from Rfam (http://rfam.janelia.org) using a BLASTN algorithm, and eventually mapped reads removed before further analysis. The remaining filtered reads were aligned against pre-miRNA and mature miRNA sequences deposited in the miRBase v. 20 (http://www.mirbase.org/ftp.shtml)^55^ using a Bowtie software^56^ with a single basepair mismatch allowed. The consensus alignments (pre-miRNA) were used as queries against the genome assembly of *A. pisum* (http://www.aphidbase.com/aphidbase/downloads) using a BLAST algorithm. Unique sequences that mapped to both insect pre-miRNAs in miRBase and to *A. pisum* genome were termed ‘conserved’ mature miRNA, whereas pre-miRNAs that mapped to insect entries in miRBase but not to *A. pisum* genome, or *vice versa*, were called “semi-conserved”. Lastly, unique sequences un-mapped to insect pre-miRNAs and *A. pisum* genome were considered as potential novel miRNAs specific for *S. avenae*. The potential hairpins formed by the flanking sequences of all putative *S. avenae* miRNA were predicted by secondary structure analysis with Mfold using the default parameters (http://mfold.rna.albany.edu/?q=mfold/RNA-Folding-Form)^57^. Criteria for miRNA annotation and secondary structure formation are listed in Table S6.

### Differential miRNA expression between winged and wingless morphs

Differences in miRNA expression were estimated using the abundance of reads from non-normalized winged and wingless *S. avenae* libraries as a proxy. The read counts of raw data output from ACGT101-miR were imported into the program IDEG6^58^ using a web portal, http://telethon.bio.unipd.it/bioinfo/IDEG6_form/. *In silico* normalization of miRNA counts between libraries was carried out based on the total number of reads across libraries. The significance of any difference in read count was assessed by Bonferroni corrected Chi-squared 2 × 2 test with a *P*-value ≤0.05 and the fold-change ≥1.5.

Nine microRNAs differentially expressed between winged and wingless *S. avenae* were validated by quantitative real-time PCR (RT-qPCR). Total RNA was extracted, respectively, from 50 mg of winged and wingless *S. avenae* adults as described above. Resultant RNA (1.0 μg) of each sample was reverse transcribed to synthesize the first strand cDNA using miScript II RT Kit (Qiagen, Germany) following manufacturer’s instructions. The miScript II RT Kit procedure attaches a universal primer at the 3′ end of cDNAs, in which a universal primer anneal size in located and used in conjunction with miRNA-specific primers for selective PCR amplification. Primers specific for *S. avenae* miRNAs were designed using Primer Premier 5.0 software (Premier Biosoft International, Palo Alto, CA, USA; Table 4) and synthesized by Sangon Biotech (Shanghai, China). The PCR amplification cycles included an initial denaturation step at 95 °C for 5 min, 35 cycles of 95 °C for 30 s, 55 °C for 30 s and 72 °C for 30 s, and a final extension step of 10 min at 72 °C. PCR products were analyzed on 2% agarose gels, stained with ethidium bromide, and visualized using the Imaging G6 System (DHS Life Science & Technology Co., Ltd., Beijing, China). All PCR products were cloned and Sanger sequenced to confirm the primer specificity for the respective sRNAs.

Quantitative real-time PCR (RT-qPCR) was conducted using the miScript SYBR^®^ Green PCR Kit (Qiagen, Germany) following manufacturer’s instructions (Table 4). The *S.avenae* microRNA *U6* was used as an endogenous small RNA reference. RT-qPCR was performed on an Applied Biosystems 7500 Real-Time PCR System under the following conditions: 95 °C for 15 min for template denaturation and 40 cycles of 94 °C for 15 s, 60 °C for 30 s and 70 °C for 34 s followed by the melting curve. Three biological replicates and three technical replications were carried out. Relative miRNA expression was calculated from cycle threshold (C_T_) data averaged across technical replicates by the comparative threshold (2^−ΔΔCT^) method^59^, and normalized across samples and replicates within samples using the microRNA *U6*. The significance of any differential expression of each candidate miRNAs between winged and wingless adults were assessed using a two sample *t*-test in SAS statistical software 9.2 (SAS Institute Inc., Cary, NC, USA), with thresholds set at a *P* < 0.05.

### miRNA target prediction

The putative mRNAs targeted by differentially-expressed *S. avenae* miRNAs were predicted against all *S. avenae* nucleotide sequences downloaded from the National Center for Biotechnology Information (http://www.ncbi.nlm.nih.gov/gene). Additionally, targets were predicted based on orthologous insect sequences from *D. melanogaster* (FlyBase; http://flybase.org/), *A. pisum* (AphidBase; http://www.aphidbase.com), and expressed sequence tags (ESTs) from *Myzus persicae*, *Toxoptera citricida* and *Aphis gosspii* (AphidBase). All fasta-formatted sequence datasets were used to create separate local databases. For the latter EST datasets, open reading frames (ORFs) were predicted using the getorf program in the EMBOSS package. ORFs were extracted using a custom PERL script and then used as quires against the NCBI nr protein database to obtain the potential CDS annotations and UTR regions. All databases were queried with the putative *S. avenae* miRNAs suing the program Target Scan with the default parameters (http://www.targetscan.org/). All identified putative target genes were used to search the Gene Ontology (GO) database and within Kyoto Encyclopedia of Genes and Genomes (KEGG) pathway analyses, with threshold set at a corrected *P*-value ≤ 0.001.

## Additional Information

**How to cite this article**: Li, X. *et al*. Comparative profiling of microRNAs in the winged and wingless English grain aphid, *Sitobion avenae* (F.) (Homoptera: Aphididae). *Sci. Rep.* **6**, 35668; doi: 10.1038/srep35668 (2016).

## Supplementary Material

Supplementary Information

Supplementary Table S1

Supplementary Table S2

Supplementary Table S3

Supplementary Table S4

Supplementary Table S5

Supplementary Table S6

## Figures and Tables

**Table 1:** Distribution of miRNA reads from winged and wingless *S. avenae.*

Parameter	Winged	%	Wingless	%
Raw reads	13,760,466	100	15,594,991	100
3ADT and length filter	769,767	43.3	911,013	44.1
Short reads	12,324	0.69	14,883	0.72
Rfam	104,957	5.9	104,038	5.04
Repeats	6,097	0.34	5,139	0.25
rRNA	42,852	0.31	46,219	0.3
tRNA	29,186	0.21	28,230	0.18
snoRNA	6,613	0.05	5,584	0.04
snRNA	7,070	0.05	6,371	0.04
Other Rfam RNA	19,236	0.14	17,634	0.11
Mappable reads	887,980	49.95	1,033,351	50.02

3ADT: reads removed due to 3ADT not found and length with <15 nt and >30 nt were removed.

Short reads: >=2N, >=7A, >=8C, >=6G, >=7T, >=10Dimer, >=6Trimer, or >=5Tetramer (N is undetermined nucleotide).

Rfam: Collection of many common non-coding RNA families except micro RNA.

Repeats: Prototypic sequences representing repetitive DNA from different eukaryotic species.

Mappable reads: reads that were passed through a series of the digital filters from the raw reads.

**Table 2:** The different expression of miRNAs in *S.avenae* small RNA libraries.

miR_Name	Norm. Reads		*P*-value	FC (WL/W)	Log_2_ (FC)	WL
	WL	W				
*PC-3p-94006_17*	1	21	6.66E − 05	21.00	−4.39	down
*sav-mir-100-p3*	1	11	7.31E − 03	11.00	−3.46	down
*PC-3p-420630_4*	1	9	1.91E − 02	9.00	−3.17	down
*sav-miR-277*^*#*^	2863	24006	0.00E + 00	8.38	−3.07	down
*PC-5p-113190_15*^*#*^	19	3	2.15E − 04	6.33	2.66	up
*sav-miR-996*	3	13	2.37E − 02	4.33	−2.12	down
*sav-miR-100*	3107	11992	0.00E + 00	3.86	−1.95	down
*sav-let-7*^*#*^	1895	6399	0.00E + 00	3.38	−1.76	down
*PC-3p-131984_11*	22	8	3.83E − 03	2.75	1.46	up
*PC-3p-40838_47*	9	21	4.80E − 02	2.47	−1.30	down
*sav-miR-1*^*#*^	337	802	5.91E − 32	2.38	−1.25	down
*sav-mir-3031-p5*	35	16	1.95E − 03	2.19	1.13	up
*sav-mir-3020-p3*	17	8	3.52E − 02	2.13	1.09	up
*PC-3p-66379_27*	32	16	6.28E − 03	2.00	1.00	up
*sav-miR-278*	1097	2118	2.06E − 48	1.93	−0.95	down
*sav-miR-3030*	26	14	2.24E − 02	1.86	0.89	up
*sav-miR-210*	141	253	8.49E − 06	1.79	−0.84	down
*PC-3p-80125_21*	22	13	4.82E − 02	1.76	0.82	up
*PC-3p-2743_844*^*#*^	622	357	3.05E − 25	1.74	0.80	up
*sav-mir-3033-p5*	33	57	4.99E − 02	1.73	−0.79	down
*sav-miR-92a*	362	622	1.48E − 10	1.72	−0.78	down
*sav-mir-92a-1-p5*	39	66	4.34E − 02	1.69	−0.76	down
*sav-mir-316-p3*	205	123	1.78E − 08	1.67	0.74	up
*sav-miR-3041*	85	54	8.15E − 04	1.57	0.65	up
*sav-miR-124*	1541	2398	4.47E − 23	1.56	−0.64	down
*sav-miR-2765*	545	351	9.94E − 17	1.55	0.63	up
*sav-miR-92b*	3549	5381	6.21E − 43	1.52	−0.60	down
*sav-miR-3016*	159	105	1.61E − 05	1.51	0.60	up
*sav-miR-315*^*#*^	2717	1857	1.06E − 63	1.46	0.55	up^*^
*sav-miR-9a*^*#*^	1177	1084	1.31E − 06	1.09	0.12	up^*^
*sav-miR-8*^*#*^	15752	14611	1.58E − 66	1.08	0.11	up^*^
*sav-miR-7*^*#*^	3489	3284	7.08E − 14	1.06	0.09	up^*^

W and WL respectively for winged and wingless morphs; FC = Fold-change; *The expression of these miRNAs showed no significance difference between winged and wingless S.avenae; ^#^RT- qPCR validation.

**Table 3:** miRNAs associated with wing development in insects.

miRNA	Role or target genes	Insects	References
*miR-9a*	*dLMO*, *senseless*, affecting wing tissue and the ectopic apoptosis	Drosophila	20
*miR-12, -283*	*Cos-2*, *fu*, *smo*, affecting *Hg* signaling pathway		25
*miR-8*	*Dll*, *se*, involving *Wnt* signaling		60
*Let-7*	*Abrupt*, stimulating cell proliferation and preventing apoptosis		18
*miR-315*	activating *wingless* signaling		47
*miR-7*	Regulating *Notch* signal		61
*miR-iab-4-5p*	*Ultrabithorax*, Wing/halter sepcification		31
*miR-iab-8-5p*	*Ultrabithorax*, *abdominal A*, Wing/halter sepcification		62
*bantam*	*Hid*, *enaled*, *mei-p26*, suppression imaginal discs		63
*miR-252-5p, miR-982-5p*	*Dis3,* suppressing wing development		29
*miR-263, -184*	affecting lepidopteron wing scale cell patterning	Butterfly	21
*miR-193, -2788*	predicting their specific functions in butterfly wing		21
*miR-2768*	*Cubitus interruptus*, involving wing primordia patterning		64
*miR-1*	Probably relating muscle development	Locust	40
*miR-125*	Predicting to regulate two phase of locust		40
*Let-7, miR-100, -125*	Affecting wing morphogenesis	Cockroach	19

**Table 4:** Primers used for RT-qPCR analysis.

Name	miRNA name	Primers
1	*miR-315*	TTTTGATTGTTGCTCAGAAAGCC
2	*miR-1*	GGAATGTAAAGAAGTATGGAG
3	*miR-9a*	TCTTTGGTTATCTAGCTGTAT
4	*PC-5p-113190_15*	TTGGATGCCTATGTGG
5	*PC-3p-2743_844*	ACAGCAAAGTGAAAGAGACTGA
6	*miR-7*	TGGAAGACTAGTGATTTTGTTGTT
7	*miR-8*	TAATACTGTCAGGTAATGATGTC
8	*miR-277*	TAAATGCACTATCTGGTACGACA
9	*Let-7*	TGAGGTAGTTGGTTGTATAGT
References	*U6*	CGCAAGGATGACACGCAA
