# Supplementary material for: Comparative profiling of microRNAs in the winged and wingless English grain aphid, Sitobion avenae (F.) (Homoptera: Aphididae)
Source: Sci Rep. 2016 Oct 20;6:35668. doi: 10.1038/srep35668 (PMC5071838; doi:10.1038/srep35668)
Supplement: Supplementary Information [file srep35668-s1.pdf]

Supplementary Materials

Comparative profiling of microRNAs in the winged and wingless English grain aphid, *Sitobion avenae* (F.) (Homoptera: Aphididae)

Xiangrui Li[1], Fangmei Zhang [1,2],Brad Coates[3], Yunhui Zhang[1], Xuguo Zhou[4,*], Dengfa Cheng[1,*]

[1]*State Key Laboratory for Biology of Plant Diseases and Insect Pests, Institute of Plant Protection, Chinese Academy of Agricultural Sciences, Haidian District, Beijing 100193, China.*
[2] *Xinyang Agriculture and Forestry University, Xinyang, Henan 464000, China.*
[3] *United States Department of Agriculture, Corn Insects & Crop Genetics Research Unit, USDA-ARS, Ames, IA 50011, USA.*
[4] *Department of Entomology, University of Kentucky, Lexington, KY 40546, USA.*

*** Corresponding Authors:**
Dr. Dengfa Cheng
Institute of Plant Protection
Chinese Academy of Agricultural Sciences
Yuanmingyuan West Road
Beijing 100193, China
Phone: 86-10-62815935
Email: dfcheng@ippcaas.cn

Dr. Xuguo "Joe" Zhou
Department of Entomology
University of Kentucky
S-225 Agricultural Science Center North
Lexington, KY40546-0091
Phone: 859-257-3125
Fax: 859-323-1120
Email: xuguozhou@uky.edu

Table Legends

Table S1. Summary of conserved miRNA.

Table S2. Summary of predicted miRNA.

Table S3. The predicted targets for *S. avenae* miRNAs based on sequences from a model species *D. melanogaster* and four aphid species,*A. pisum, M. persicae, T. citricida,* and *A. gosspii*.

Table S4. GO annotation for identified *S. avenae* miRNAs.

Table S5. KEGG analysis for targets of differentially expressed *S. avenae* miRNAs.

Table S6. Criteria used for miRNA prediction.

Figure Legends

Figure S1. Homology analysis of *S. avenaeLet-7* with other insects. api: *Acyrthosiphon pisu*m; bmo: *Bombyx mori*; tca: *Tribolium castaneum*; der: *Drosophila erecta*; dpe: *Drosophila persimilis*; dan: *Drosophila ananassae*; dwi: *Drosophila will*; isc: *Ixodes scapularis*; ame: *Apis mellifera*;cqu: *Culex quinquefasciatus*; dmo: *Drosophila mojavensis*; mse: *Manduca sexta*; dgr: *Drosophila grimshawi*; aga: *Anopheles gambiae*; dme: *Drosophila melanogaster*; dvi: *Drosophila virilis*; aae: *Aedes aegypti*; hme: *Heliconius Melpomene*; tur: *Tetranychus urticae*; dya: *Drosophila yakuba*; dse: *Drosophila sechellia*; ngi: *Nasonia giraulti*; dsi: *Drosophila simulans*; nlo: *Nasonia longicornis*; nvi: *Nasonia vitripennis.* (The red box indicates the miRNA seedpositions 2-8)

Figure S2. Homology analysis of *S. avenaemiRNA-7* with other insects. api: *Acyrthosiphon pisu*m; bmo: *Bombyx mori*; tca: *Tribolium castaneum*; der: *Drosophila erecta*; dpe: *Drosophila persimilis*; dan: *Drosophila ananassae*; dwi: *Drosophila will*; isc: *Ixodes scapularis*; ame: *Apis mellifera*;cqu: *Culex quinquefasciatus*; dmo: *Drosophila mojavensis*; mse: *Manduca sexta*; dgr: *Drosophila grimshawi*; aga: *Anopheles gambiae*; dme: *Drosophila melanogaster*; dvi: *Drosophila virilis*; aae: *Aedes aegypti*; hme: *Heliconius Melpomene*; tur: *Tetranychus urticae*; dya: *Drosophila yakuba*; dse: *Drosophila sechellia*; ngi: *Nasonia giraulti*; dsi: *Drosophila simulans*; nlo: *Nasonia longicornis*; nvi: *Nasonia vitripennis.* (The red box indicates the miRNA seedpositions 2-8)

Figure S3.Predicted stem-loop structures for three novel miRNA precursors.

Figure S4. PCR Products of nine selected miRNAs potentially involved in *S. avenae* wing development. cDNAs from the winged *S. avenae* were used for template. **Lane 1**: 100bp ladder marker; **Lane 2**: *miR-315*; **Lane 4**: *miR-1*; **Lane 6**: *miR-9a*; **Lane 8**: *PC-5p-113190_15*; **Lane 10**: *PC-3p-2743_844*; **Lane 12**: *miR-7*; **Lane 14**: *miR-8*; **Lane 16**: *miR-277*; **Lane 18**: *Let-7*. The other uneven lanes were negative controls for each target miRNA.

Figures

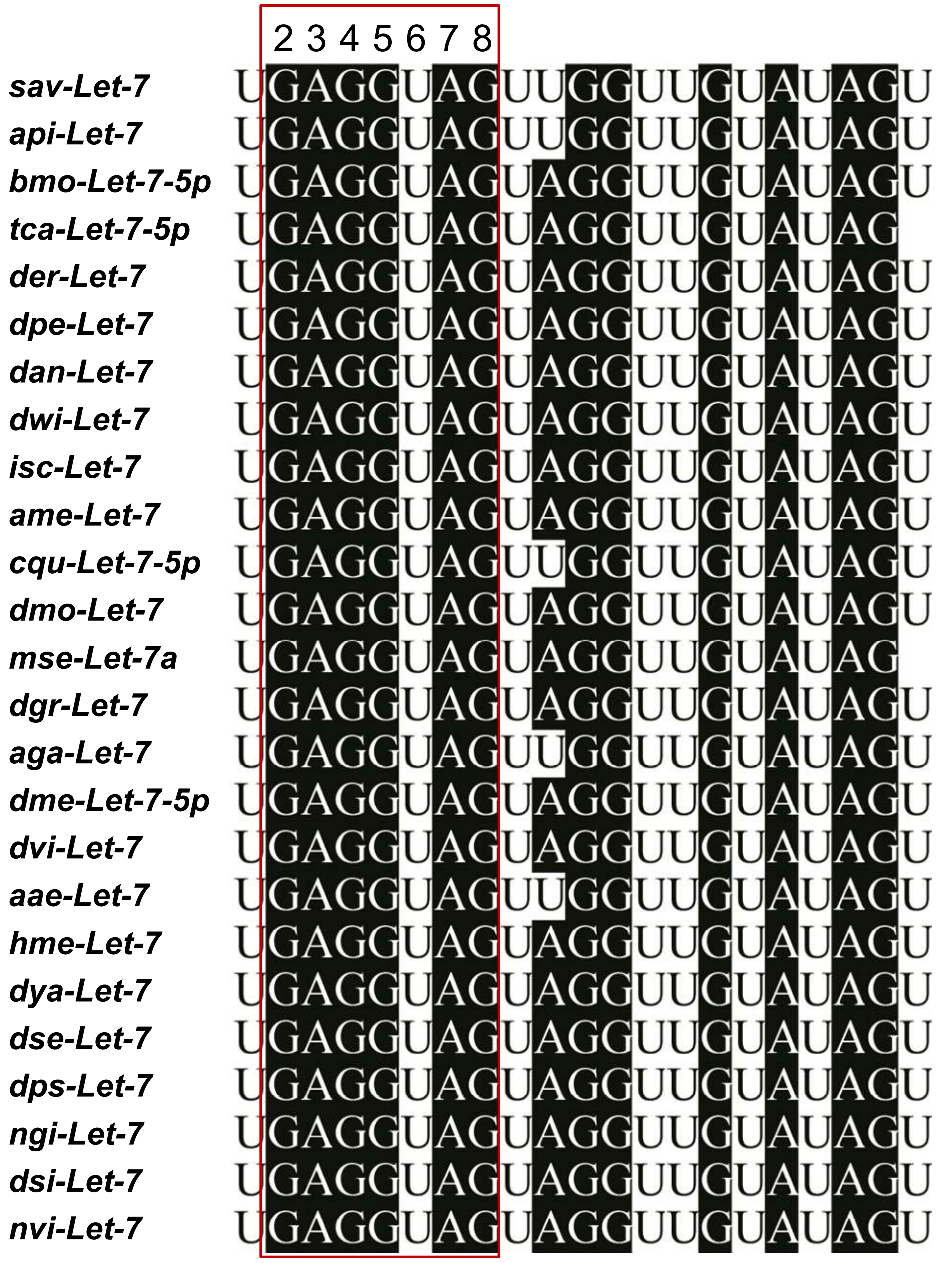

Figure S1

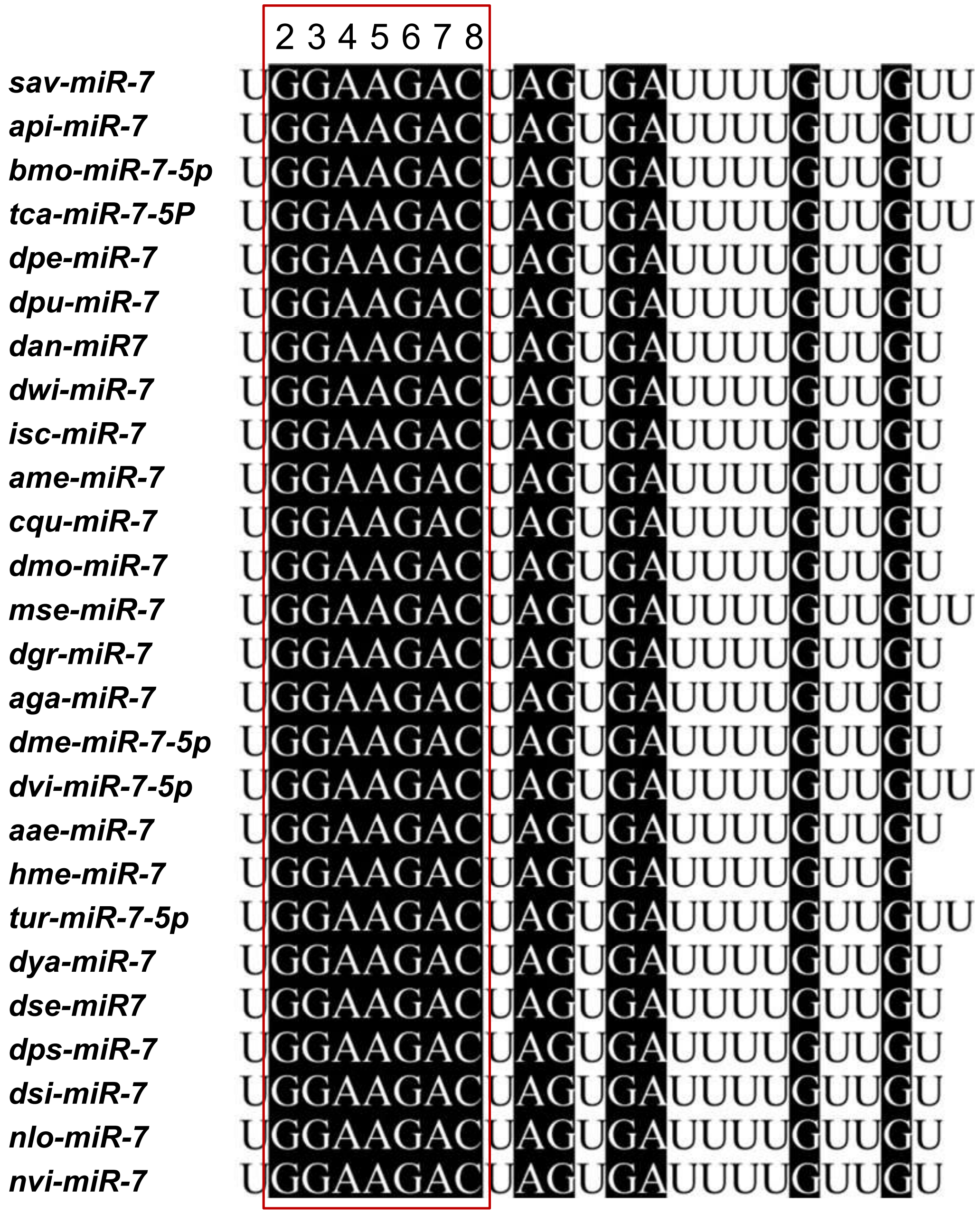
2 3 4 5 6 7 8
sav-miR-7 UGGAAGACUAGUGAUUUUGUUGUU
api-miR-7 UGGAAGACUAGUGAUUUUGUUGUU
bmo-miR-7-5p UGGAAGACUAGUGAUUUUGUUGU
tca-miR-7-5P UGGAAGACUAGUGAUUUUGUUGUU
dpe-miR-7 UGGAAGACUAGUGAUUUUGUUGU
dpu-miR-7 UGGAAGACUAGUGAUUUUGUUGU
dan-miR7 UGGAAGACUAGUGAUUUUGUUGU
dwi-miR-7 UGGAAGACUAGUGAUUUUGUUGU
isc-miR-7 UGGAAGACUAGUGAUUUUGUUGU
ame-miR-7 UGGAAGACUAGUGAUUUUGUUGU
cqu-miR-7 UGGAAGACUAGUGAUUUUGUUGU
dmo-miR-7 UGGAAGACUAGUGAUUUUGUUGU
mse-miR-7 UGGAAGACUAGUGAUUUUGUUGUU
dgr-miR-7 UGGAAGACUAGUGAUUUUGUUGU
aga-miR-7 UGGAAGACUAGUGAUUUUGUUGU
dme-miR-7-5p UGGAAGACUAGUGAUUUUGUUGU
dvi-miR-7-5p UGGAAGACUAGUGAUUUUGUUGUU
aae-miR-7 UGGAAGACUAGUGAUUUUGUUGU
hme-miR-7 UGGAAGACUAGUGAUUUUGUUG
tur-miR-7-5p UGGAAGACUAGUGAUUUUGUUGUU
dya-miR-7 UGGAAGACUAGUGAUUUUGUUGU
dse-miR7 UGGAAGACUAGUGAUUUUGUUGU
dps-miR-7 UGGAAGACUAGUGAUUUUGUUGU
dsi-miR-7 UGGAAGACUAGUGAUUUUGUUGU
nlo-miR-7 UGGAAGACUAGUGAUUUUGUUGU
nvi-miR-7 UGGAAGACUAGUGAUUUUGUUGU

Figure S2

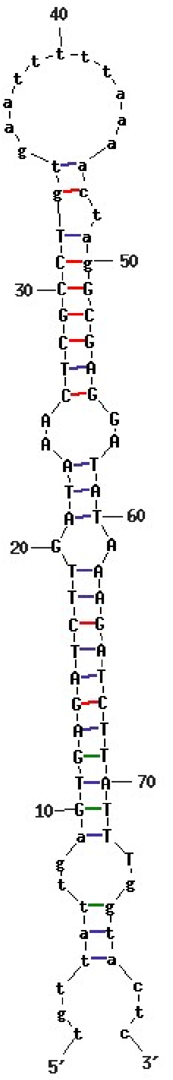

PC-3p-99145_16

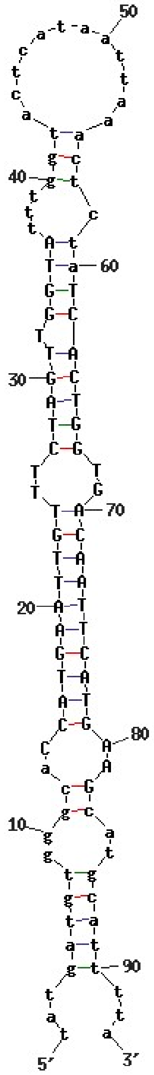

PC-3p-55615_34

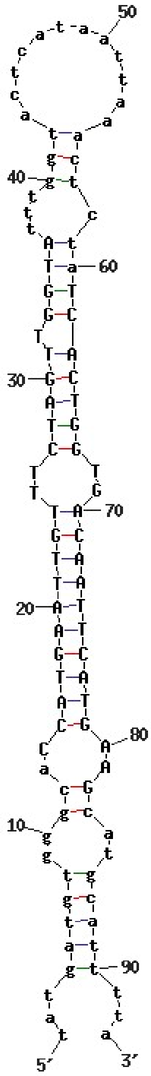

PC-5p-373819_3

Figure S3

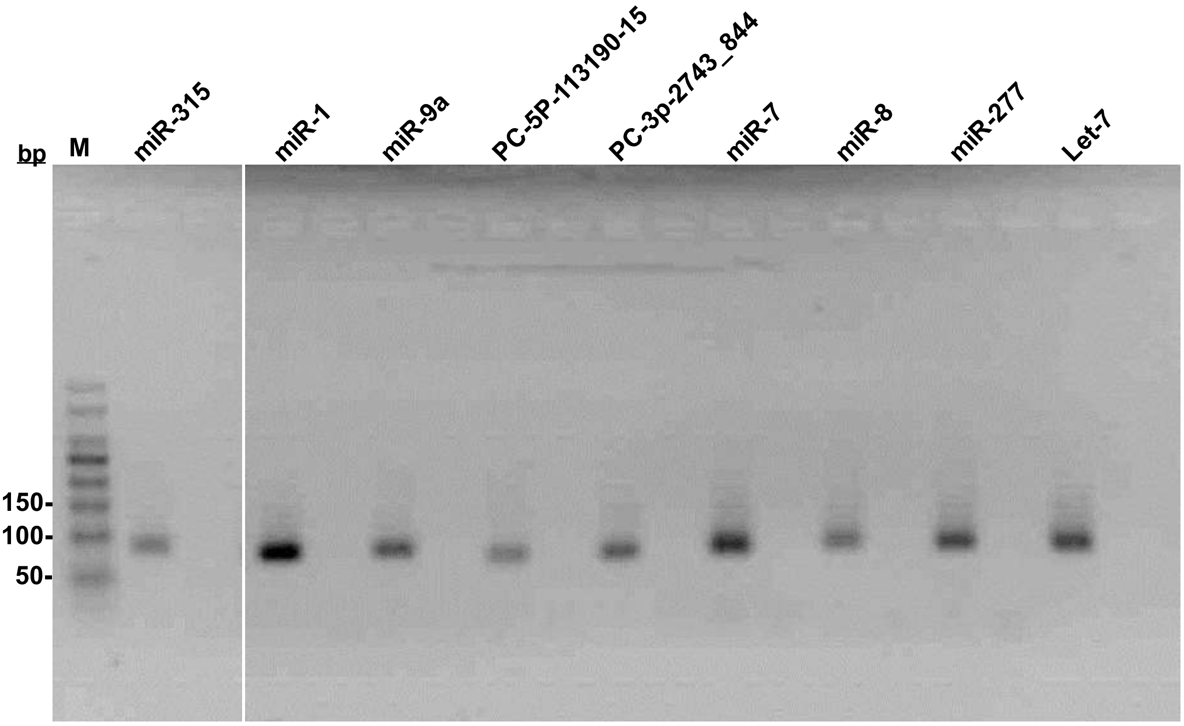
bp
M
miR-315
miR-1
miR-9a
PC-5P-113190-15
PC-3p-2743_844
miR-7
miR-8
miR-277
Let-7
150-
100-
50-

Figure S4
